# Quantitative impacts of incubation phase transmission of foot-and-mouth disease virus

**DOI:** 10.1038/s41598-019-39029-0

**Published:** 2019-02-25

**Authors:** Jonathan Arzt, Matthew A. Branan, Amy H. Delgado, Shankar Yadav, Karla I. Moreno-Torres, Michael J. Tildesley, Carolina Stenfeldt

**Affiliations:** 1 Foreign Animal Disease Research Unit, Plum Island Animal Disease Center, Agricultural Research Service, United States Department of Agriculture, Greenport, NY USA; 2 Monitoring and Modeling, Center for Epidemiology and Animal Health, Animal and Plant Health Inspection Service, United States Department of Agriculture, Fort Collins, CO USA; 3 PIADC Research Participation Program, Oak Ridge Institute for Science and Education, Oak Ridge, TN USA; 4 Zeeman Institute (SBIDER), School of Life Sciences and Mathematics Institute, University of Warwick, Coventry, UK; 5 Department of Veterinary Population Biology, University of Minnesota, St. Paul, MN USA

## Abstract

The current investigation applied a Bayesian modeling approach to a unique experimental transmission study to estimate the occurrence of transmission of foot-and-mouth disease (FMD) during the incubation phase amongst group-housed pigs. The primary outcome was that transmission occurred approximately one day prior to development of visible signs of disease (posterior median 21 hours, 95% CI: 1.1–45.0). Updated disease state durations were incorporated into a simulation model to examine the importance of addressing preclinical transmission in the face of robust response measures. Simulation of FMD outbreaks in the US pig production sector demonstrated that including a preclinical infectious period of one day would result in a 40% increase in the median number of farms affected (166 additional farms and 664,912 pigs euthanized) compared to the scenario of no preclinical transmission, assuming suboptimal outbreak response. These findings emphasize the importance of considering transmission of FMD during the incubation phase in modeling and response planning.

## Introduction

Preparedness for infectious disease outbreaks can be greatly enhanced by the availability of models that can predict the transmission of pathogens and assess the potential effectiveness of control measures^1,2^. The reliability of such models depends upon elucidation of key epidemiologic parameters for the disease, as well as a strong understanding of the transmission dynamics in susceptible populations and the effect of intervention strategies on preventing disease spread^3^. A variety of models have been developed in recent years to identify critical targets for control efforts, predict impacts, and estimate resource requirements for specific outbreak scenarios for foot-and-mouth disease (FMD)^4^. However, it is critical that mathematical models applied to predict disease spread and impact are built upon parameters that are reliable and representative for the specific host-pathogen combination^2^. In addition, models used to inform disease control planning efforts need to be able to adequately represent the complexity of the livestock production systems involved and the often inter-linked and overlapping disease control strategies likely to be deployed. Similarly, thorough understanding of the biological relevance of modeled parameters is needed for appropriate interpretation of the modeled output^5,6^. With increasing emphasis on applying disease control measures in a manner that minimizes the impact and disruption to non-affected industries, models used to inform national strategies for FMD control must often address complex aspects of farm structure, demography, and animal movements, as well as surveillance, quarantine, and depopulation strategies^7,8^. While the phenomenon of incubation phase transmission of FMD in pigs has been previously identified^9^, the impact of preclinical transmission and the value of explicitly including this phase in disease spread and control modeling has not been evaluated.

**Figure 1:**
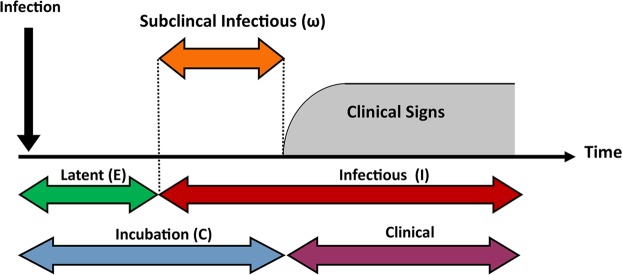
Definitions used to characterize distinct periods of infectious diseases. The latent period (E; green arrow) begins at the time of infection and ends at the onset of the infectious period. (I; red arrow). The incubation period (C; blue arrow) starts at the time of infection and ends at the appearance of clinical signs of disease (purple arrow). The difference between incubation and latency is denoted by ω (omega), and can have either a positive or negative value depending on whether transition to the infectious period occurs before or after appearance of clinical signs. The ratio of transmission occurring during incubation, and transmission occurring through the total infectious period is denoted by θ (theta; not shown).

The classical definitions of disease state progressions at the level of the infected individual includes the incubation (pre-clinical) and the latent (pre-infectious) periods. Disease control becomes increasingly challenging when the latent period is shorter than the incubation period, since an infected individual may transmit the pathogen to numerous contacts prior to identification of clinical disease and subsequent implementation of control interventions^10^. The proportion of transmission that occurs during the incubation phase is commonly denoted by theta (θ)^11,12^. A low value of θ indicates correspondingly low extent of transmission prior to observation of clinical disease. Although a low θ is relatively uncommon, this has been associated with infectious diseases for which control or eradication programs have been highly successful such as smallpox and rinderpest^11,13^. At the other end of the spectrum are diseases for which θ is remarkably high, such as HIV/AIDS, which are notoriously difficult to control^11^. Closely related to the concept of θ is the temporal disparity between incubation and latency which represents the period of infectiousness included within the incubation period, defined herein as ω (omega; Fig. 1). While θ is intrinsically linked to the total length of the infectious period (often difficult to measure), ω can be estimated without having knowledge of the termination of infectiousness. Additionally, the basic reproduction number (R_0_) of a pathogen is frequently cited to project and characterize an infectious disease epidemic. This quantity is defined as the average number of secondary cases generated by a typical primary case during its entire period of infectiousness, in a completely susceptible population and in the absence of control measures^14,15^. Thus, in addition to aspects intrinsic to the specific host-pathogen combination, R_0_ will also be affected by numerous extrinsic factors at the population level. Combining estimates for R_0_, θ, and ω provides a robust background for evaluating the importance of preclinical transmission in successful modeling of disease spread and control interventions.

This current study focused on investigating the concept of θ and ω for direct contact transmission of a virulent strain of foot-and-mouth disease virus (FMDV) amongst juvenile domestic pigs, and examining the impact of pre-clinical transmission on simulated outbreak size and severity within a US swine production system assuming either optimal or suboptimal response conditions. FMDV is a highly contagious viral pathogen of cloven-hoofed domestic and wild animals (genus: *Aphthovirus*, family: *Picornaviridae*)^16,17^. The virus, which is the causative agent of foot-and-mouth disease (FMD), has been associated with multiple major disease epidemics in previously free regions in recent years^18–20^. Given the highly contagious nature of the disease, the ability to predict potential FMD dissemination in a naïve population and assess the effectiveness of control interventions can result in critical improvements in preparedness and emergency response for food animal production systems^4^. However, data-driven modeling is intrinsically and precariously dependent upon the appropriateness of the data used for parameterization. Recent work examining incubation phase transmission of FMDV amongst cattle has been inconsistent between two studies suggesting either a high value of θ for dairy cattle or a low value for calves^12,21^. In contrast to this, a similar investigation demonstrated the occurrence of substantial transmission of FMDV during the incubation phase in group-housed pigs^9^. This current report provides novel modeling approaches based upon the data obtained from this recent transmission study^9^. The findings presented herein suggest a difference between the duration of the latent- and incubation periods (pre-clinical infectiousness; ω) in FMDV-infected pigs that is statistically significantly greater than 0, and a resultant θ that is larger than previously suggested in studies of FMDV transmission in cattle. Additionally, simulating FMD outbreak scenarios using fixed and incrementally increasing values of ω demonstrated significant influence of incubation phase transmission on the size and duration of FMD outbreaks in US pig production systems, even in the presence of rigorous control strategies.

## Results

### FMDV transmission study

**Figure 2:**
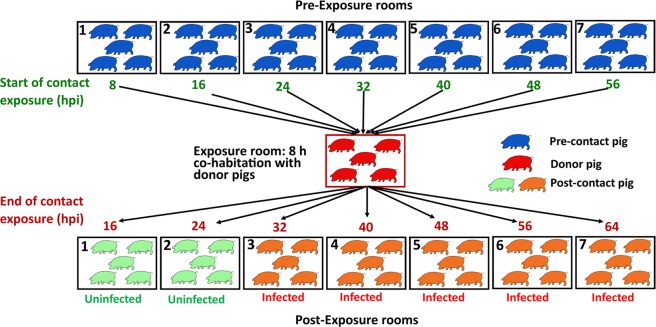
Experimental design, FMDV transmission study. Seven groups of five pigs each were exposed to five FMDV-infected donor pigs through successive eight hour exposure periods. Contact groups were housed in separate isolation rooms before and after exposure to the donor pigs. The time points in the figure represent beginning (green) and end (red) of exposure for each contact group in relation to inoculation of the donor pigs. There was no transmission of infection to contact groups 1 and 2 (exposed from 8–16, and 16–24 hours post infection of donors, respectively). All pigs in contact groups 3 through 7 developed clinical FMD after exposure to the donors.

The experimental basis for the analyses presented herein consisted of a transmission study in which groups of naïve pigs were exposed to a group of FMDV-infected “donor pigs” through successive eight hour time periods (Fig. 2)^9^. Successful transmission from donors was determined by confirmed occurrence of FMD in contact-exposed pigs subsequent to exposure. The progression of infection was monitored and quantitated in both donors and contact-exposed pigs through measurements of viral genomic RNA in serum and oropharyngeal fluids (OPF). In brief, all 5 pigs in the donor group were confirmed to have been infected by needle-free oropharyngeal inoculation with FMDV and were shedding FMDV RNA in OPF throughout the contact transmission trial. Viremia in donor pigs was first detected at 24 hours post inoculation (hpi), whereas fever (rectal temperatures >40 °C) and vesicular lesions were observed simultaneously at 48 hpi (Supplementary Fig. S1).

There were no transmission events between the donors and contact groups 1 or 2, which had been exposed to the donor pigs from 8 to 16 hpi, and 16 to 24 hpi, respectively. Contrastingly, all pigs in contact groups 3 through 7, corresponding to exposure from 24 through 64 hpi of the donors, developed clinical FMD (Fig. 2). On this basis it was experimentally demonstrated that transmission from donor pigs had occurred at least 24 hours prior to detection of clinical disease^9^.

### Modeling of infection dynamics in donors

**Figure 3:**
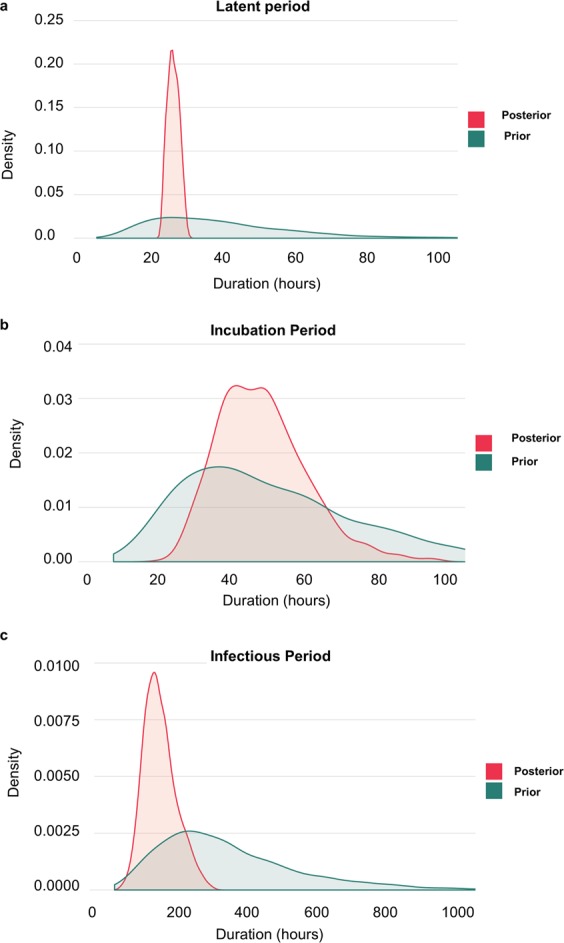
Prior and posterior distributions of latent (E), incubation (C) and infectious (I) periods in FMDV-infected donor pigs. The distribution of the latent period (**a**) was significantly updated by observations from the current investigation. The distributions of the incubation (**b**) and infectious periods (**c**) were not significantly changed.

A Bayesian model was used to estimate the length of the latent, incubation, and infectious periods of the donor pigs based on data from the transmission trial^9^. The model estimated the latent period to last slightly longer than one day (median 27 hours, 95% CI 24–30 hours) (Table 1, Fig. 3). The incubation period was estimated to be approximately 2 days (median 48 hours, 95% CI: 29–71), and the total infectious period was estimated to be approximately 7 days (median 180 hours, 95% CI: 110–270 hours). The posterior median latent period was shorter than the prior median latent period (Fig. 3a). Using the prior information, the latent period ended approximately 36 hours after inoculation. By the posterior information, the length of the latent period was updated to last about 27 hours (Table 1). Thus, the latent period is likely longer than a day and the effect of the observed data was large enough to influence the diffuse prior information (Table 1; Fig. 3a). In contrast to this, the durations of the incubation and total infectious periods by posterior distributions were not significantly different from the prior distributions (Fig. 3b,c).

**Table 1:** Posterior distribution summary statistics for parameters of interest derived from Bayesian modeling of animal level infection dynamics.

Transmission metric	Symbol	Posterior median	Posterior 95% CI
Latent period	E	27	(24, 30)
Incubation period	C	48	(29, 71)
Infectious period	I	180	(110, 270)
Subclinical infectious period	ω	21	(1.1, 45)
Proportion of total infectious period that is subclinical	*θ*	0.12	(0.00083, 0.27)
Mean length of latent period	µ_E_	27	(25, 30)
Mean length of incubation period	µ_C_	48	(29, 74)
Mean length of infectious period	µ_I_	180	(110, 280)
Transmission rate	β	27	(13, 57)

Posterior estimates for the latent (E), incubation (C), and infectious (I) periods and their means (μ_E,_ μ_C,_ and μ_I_) are expressed in terms of hours for a typical member of the donor animal group. Parameter estimates for *θ* are in terms of a proportion, and β is expressed in terms of transmission rate.

A specific interest of this part of the analysis was to determine the duration of infectiousness during the incubation period (ω) as well as the proportion of transmission that occurred during this subclinical infectious period relative to total transmission (θ). The estimation of θ relies on the length of the total infectious period, which is comprised by both subclinical and clinical phases. Assuming a median total infectious period of 180 hours as estimated by the Bayesian model, θ was estimated to be 0.12. The duration of the subclinical phase of infectiousness corresponds to the difference between incubation and latent periods, both of which were modeled based upon directly measured experimental data. Given the posterior medians of the latent (27 hours) and incubation (48 hours) periods, the difference between these values indicates a median duration of subclinical infectiousness (ω) of approximately 21 hours (95% CI: 1.1–45.0 hours; Table 1).

### Estimating the sensitivity of θ to variations in duration of infectious period

Theta (θ) represents the fraction of transmission that occurs during the incubation phase in relation to transmission occurring through the total infectious period and is thereby intimately linked to the duration of the total infectious period. Yet, no study has effectively measured the duration of infectiousness in FMDV-infected animals experimentally; rather, modeling studies have universally used proxies to generate estimates for this variable. In order to explore the sensitivity of θ to variations of the infectious period, θ was modeled using step-wise increasing durations of infectiousness ranging from 1 to 14 days, while keeping ω at the modeled estimate of 26.7 hours. The maximum resultant value of θ, which was based on modeling an infectious period of 1 day was 0.54 (95% CI 0.05–1.00; Supplementary Table S1). Contrastingly, if modeling a duration of infectiousness of 14 days, the resulting estimate of θ was 0.0055 (95% CI 0.0020–0.14; Supplementary Table S1). As expected, θ varied inversely with duration of infectiousness throughout the sensitivity analysis.

### Modeling infection dynamics using different proxy measures for onset of infectiousness

Because transmission is rarely directly quantified in experimental or field studies, we tested the ability of our Bayesian model to characterize transmission using various forms of more commonly available proxy measures for transmission. For this purpose, four different proxy measures were assessed for their ability to predict the onset of infectiousness in FMDV-infected pigs. The four evaluated proxies were (a) detection of FMDV RNA in serum, (b) detection of any FMDV RNA in OPF, (c) detection of FMDV RNA in OPF above a threshold of 6.5 log_10_ genome copy numbers (GCN)/ml, (d) clinical signs of FMD defined by the first occurrence of vesicular lesions (Table 2).

**Figure 4:**
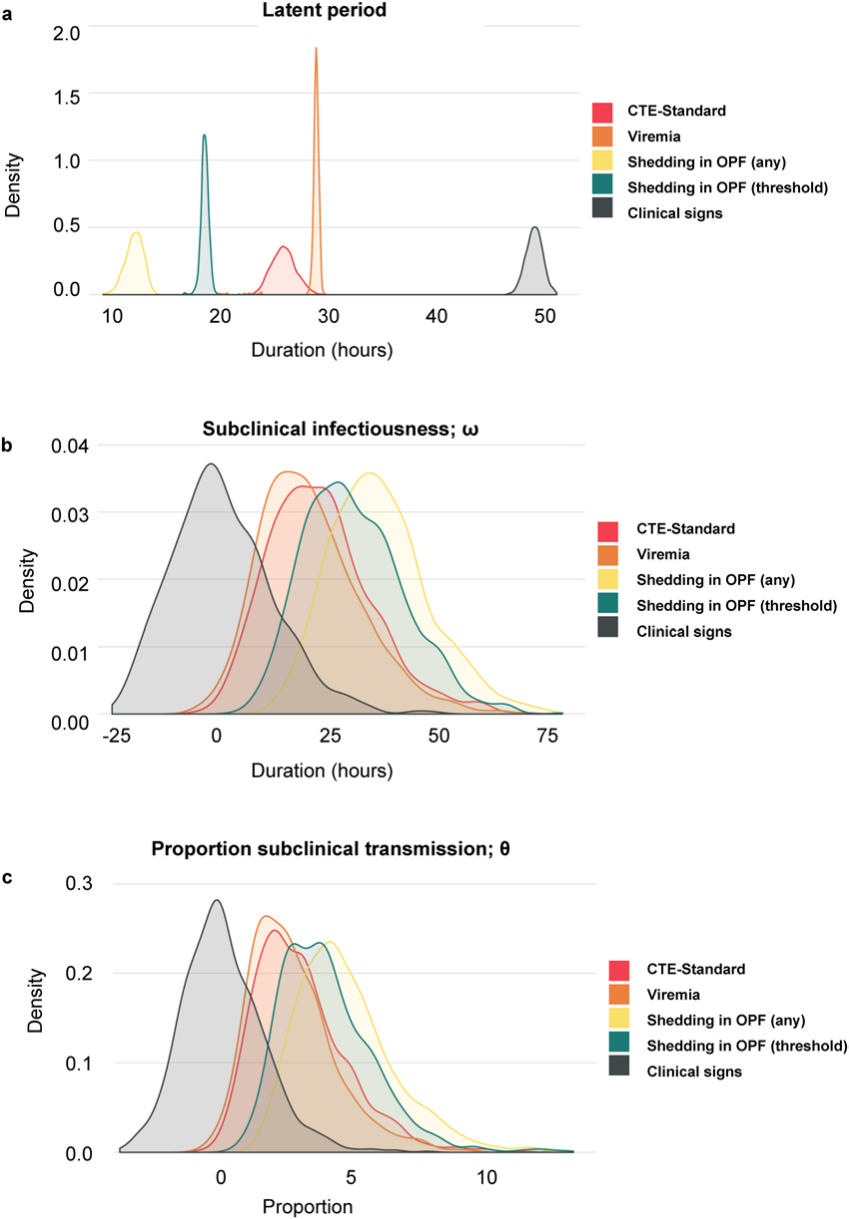
Posterior densities of durations of latency and subclinical infectiousness, and the proportion of subclinical transmission using different proxies for transmission. Four different proxy measures of contagiousness were compared to confirmed transmission events (“CTE-standard”) to model infection dynamics in FMDV infected pigs. The proxy measures consisted of detection of FMDV RNA in blood (“Viremia”), detection of FMDV shedding in oropharyngeal fluid (OPF) either above the assay lower limit detection (“any” shedding), or above a defined threshold of 6.5 log_10_ FMDV RNA copies per ml (“threshold” shedding), or detection of clinical signs of FMD. The duration of latency (**a**) was underestimated compared to the CTE-standard, when using FMDV shedding in OPF (“any” or “threshold” shedding) to define the onset of infectiousness. Detection of viremia as a proxy of infectiousness led to an estimated latent duration that was close to the CTE-standard, whereas defining infectiousness by detection of clinical signs overestimated the duration of latency. The duration of subclinical infectiousness (**b**) and the proportion of transmission during the incubation phase (**c**) were underestimated when the onset of infectiousness was based on detection of clinical signs. The estimates based on the remaining four proxy-measures were less dispersed. For both parameters, detection of viremia provided the estimates closest to the CTE-standard, whereas detection of FMDV shedding in OPF provided slightly higher estimates.

In order to test the reliability of these proxies to predict transmission, the Bayesian model of donor pig infection dynamics was repeated to compare the model outcomes when the onset of infectiousness in donor pigs was defined based on the proxy measures rather than confirmed transmission events (CTE-standard). The most noteworthy findings from this approach were the substantial changes in the modeled estimates of the duration of latency and subclinical infectiousness (ω) when defining the onset of infectiousness by either the occurrence of clinical signs of FMD in donor pigs or by any detection of FMDV RNA in OPF. Defining the onset of infectiousness by the occurrence of clinical signs in donors led to a 23 hour discrepancy of latency compared to the CTE-standard, with a prolonged latent period of 50 hours (95% CI 48–53 hours), and an ω of −1.20 hours (Fig. 4, Supplementary Table S2). Similarly, detection of (any) FMDV RNA in OPF as indicator of infectiousness resulted in a shortened latent period lasting only 13 hours (95% CI 11–15 hours) and a resultant duration of subclinical infectiousness (ω) of 35 hours (95% CI 15–59 hours; Fig. 4, Supplementary Table S2). Defining the onset of infectiousness by detection of FMDV RNA in OPF above a threshold of 6.50 log_10_ GCN/ml led to marginally decreased durations of latency and ω, whereas use of the proxy, detection of FMDV in serum led to the closest estimate to the CTE-standard (Fig. 4, Supplementary Table S2).

**Table 2:** Proxy measures used to determine the onset of infectiousness.

Transmission metric	Criteria	Units of measurement
Serum	Any virus RNA in serum measurement	log_10_ GCN/ml
FMDV in OPF (baseline)	Any measurement of virus in OPF after 8 hours exceeding the baseline limit of detection (3.08 log10 GCN/ml)	log_10_ GCN/ml
FMDV in OPF (threshold)	Any measurement of virus in OPF after 8 hours exceeding 6.50 log10 GCN/ml	log_10_ GCN/ml
Clinical signs	Any positive clinical lesion score	Numeric values between 0 and 5, with 0.25 increments corresponding to observations of lesions in donor pigs
Confirmed Transmission Event (CTE) - Standard	Confirmed transmission of FMDV to contact-exposed pigs	Numeric values between 0 and 5, with 0.25 increments corresponding to observations of lesions in contact-exposed pigs

**Table 3:** Prior distributions and estimates used to model animal level infection dynamics.

Transmission metric	Symbol	Prior distribution	Prior median	Prior 95% CI
Latent period	E	LogNormal(µ_E_, σ_E_)	36	(18, 72)
Incubation period	C	LogNormal(µ_C_, σ_C_)	48	(24, 96)
Infectious period	I	LogNormal(µ_I_, σ_I_)	180	(110, 300)
Mean length of latent period	µ_E_	Normal(log(1.5), 0.05)	36	(33, 40)
Mean length of incubation period	µ_C_	Normal(log(2), 0.05)	48	(44, 53)
Mean length of infectious period	µ_I_	Normal(log(7.5), 0.05)	180	(160, 200)
Standard deviation of latent period	σ_E_	34	34	(34, 34) [fixed]
Standard deviation of incubation period	σ_C_	34	34	(34, 34) [fixed]
Standard deviation of infectious period	σ_I_	31	31	(31, 31) [fixed]
Correlation coefficient between the latent and infectious periods	*ρ*_*EC*_	0.00	0.00	(0.00, 0.00) [fixed]
Transmission rate	β	Exponential(1/100)	68	(2.7, 370)

Posterior estimates for the latent (E), incubation (C), and infectious (I) periods, their means (μ_E,_ μ_C,_ and μ_I_), and their standard deviations (σ_E_, σ_C_, and σ_I_) are expressed in terms of hours for a typical member of the donor animal group. Parameter estimates for *θ* are in terms of a proportion, and β is expressed in terms of transmission rate.

### Effect of subclinical infectiousness (ω) on magnitude of simulated FMD outbreak scenarios

Our Bayesian modeling of infection dynamics in FMDV-infected pigs estimated the occurrence of a subclinical infectious period (ω) of 21 hours (Table 1). The practical ramification of this finding is that pigs that are infected with FMDV are capable of transmitting disease for approximately 1 day prior to the development of any visible signs of infection, which could lead to further disease spread through animal movements and indirect contacts before a producer realizes through clinical observation that there is a health problem in the herd. In order to assess the impact of the duration of the subclinical infectious period in FMDV-infected pigs on outbreak size and duration, we performed a series of FMD outbreak simulations with the US national FMD model (InterSpread Plus (ISP) version 6.0 model software^22^), which can account for the complex movements and interactions seen in intensive pig production systems. Simulations were run on a reduced farm population file composed of 45,509 swine operations with 8 different production types and a total of 54,628,373 pigs, in the eastern United States (Supplementary Fig. S2). Surveillance and control measures were modeled including passive and active surveillance in zones around infected premises, movement restrictions in the 10k zone around infected premises, and depopulation of infected premises. Under optimal conditions, detection of the index case was based on the onset of clinical signs within the herd and depopulation occurred within 3 to 5 days (depending on herd size) at a rate of 15 farms/day. For the suboptimal response, initial detection was delayed 14 days, and depopulation was delayed 7 days. For both the optimal and suboptimal responses, the duration of subclinical infectiousness was varied from 0 to 5 days, and the effect on outbreak size and duration examined. Overall, incremental increases of subclinical infectious period (ω) led to substantial increases in the size and severity of simulated outbreaks under both categories of outbreak responses.

**Figure 5:**
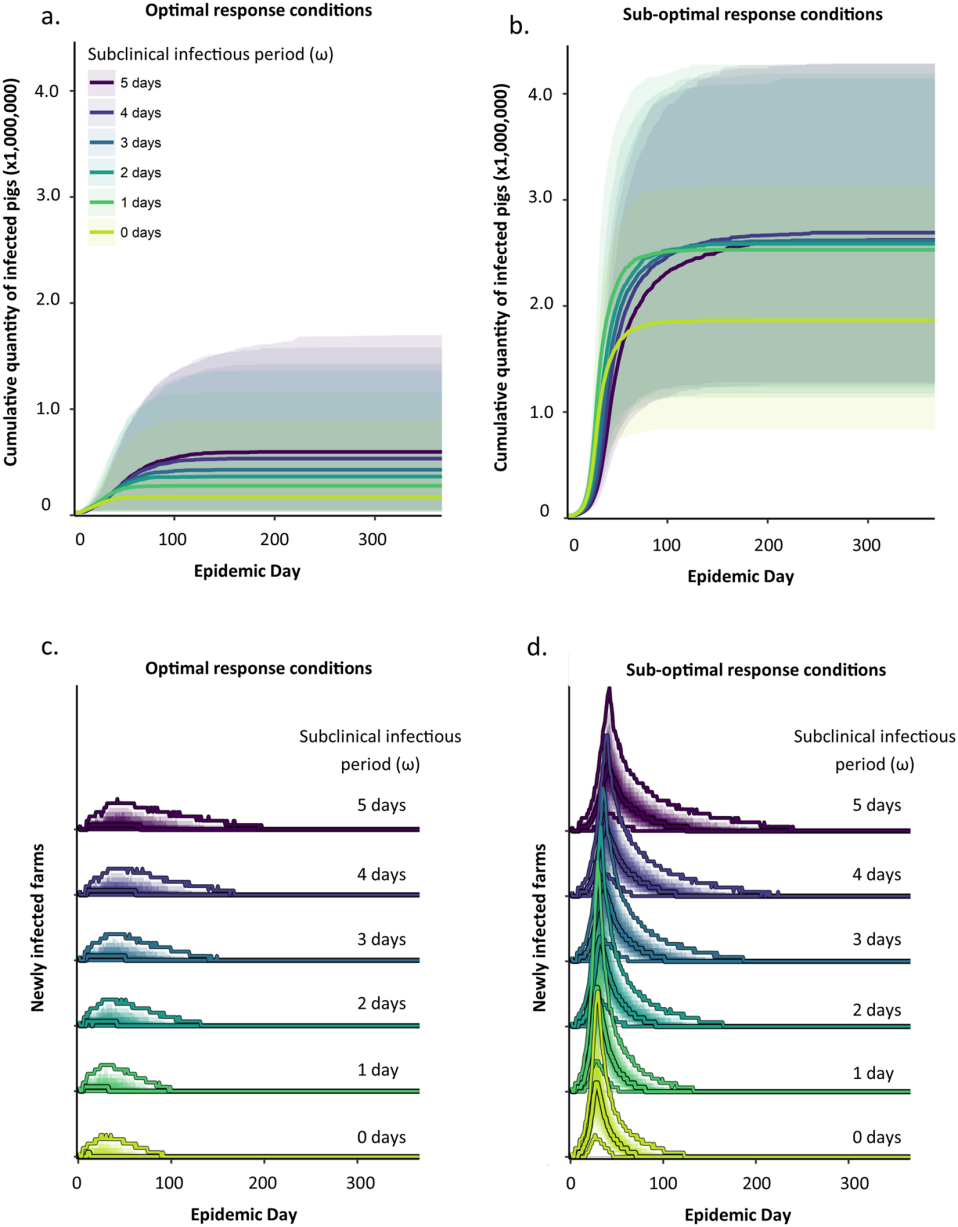
Output of FMD outbreak simulations based on increasing durations (1–5 days) of subclinical infectiousness under optimal and suboptimal outbreak response conditions. Ribbon plots for the cumulative number of infected pigs (**a**) and ridge plots for the epidemic curve (**b**) when modeled using incrementally increasing durations of subclinical infectiousness (ω) and assuming optimal (left panels) and suboptimal (right panels) outbreak responses. The lower edge, central line, and upper edge of the plots represent 5th percentile, median, and 95th percentile, respectively for the specific duration of the subclinical infectiousness (ω).

When outbreak response conditions were assumed to be optimal, a 1-day subclinical infectious period (ω = 1 day) as compared to absence of subclinical infectiousness (ω = 0 day), resulted in a 78% increase of the median number of infected farms, necessitating euthanasia of 104,394 more pigs, and an increase of the median outbreak duration from 39 to 48 days (Fig. 5 and Supplementary Table S3). When the outbreak response was assumed to be suboptimal, the effects of increasing ω were more profound, with a one-day subclinical infectious period resulting in median outbreak size increasing by 166 farms (40%), outbreak duration increasing from 88 days to 101 days, and euthanasia of 664,912 additional pigs required (Fig. 5 and Supplementary Table S3). Further increases of ω resulted in corresponding increases in outbreak size and severity, under both optimal and suboptimal outbreak response conditions (Fig. 5). Specifically, each incremental increase in omega led to a longer duration of the outbreak, with some increments having significant effect. The greatest magnitude of effect that was estimated occurred with a 5-day subclinical infectious period (ω = 5 day) combined with suboptimal outbreak response which resulted in 1.4% of farms, and 4.8% of all pigs in the population (2,622,454 pigs) becoming infected.

## Discussion

Effective control of infectious disease outbreaks is dependent upon rapid identification of infected individuals and mitigation of risks that could otherwise cause widespread dissemination of contagion. In the absence of an outbreak, data-driven mathematical models that can estimate disease spread and the impacts of control interventions can offer valuable insights to enable planning and preparedness^2^. The ultimate goal of such modeling is to guide and assess the effectiveness of control measures to minimize disease impacts, quantitated as morbidity, mortality, economic loss, or other relevant metrics^4,5^. The overarching objective of the current investigation was to use detailed data from a carefully executed experimental study of FMDV transmission^9^ to model the durations of distinct stages of disease in FMDV-infected pigs, and to highlight the importance of addressing incubation phase (subclinical) transmission in FMD models. In order to explore the concept of disease spread before clinical detection, it was of particular interest to estimate the latent period (time from infection until onset of infectiousness) in relation to the incubation period (time from infection until appearance of clinical signs of disease). The estimated parameters were subsequently used to explore the impact of alterations in the duration of subclinical infectiousness on simulated FMD outbreaks in commercial swine production systems under both optimal and suboptimal response conditions.

Disease modeling is critically dependent upon input parameters that closely reflect the intrinsic properties of the infectious agent as well as externally variable features of the modeled scenario (e.g. population composition and density, contact networks and patterns, topographical characteristics, meteorological variations and availability of resources)^2,23^. The simulations in this current investigation utilized the national FMD model developed by the US Department of Agriculture to simulate FMD outbreaks within the US pig production system. This model is adapted to specific conditions within the US agricultural system, and parameterized to reflect the complex and overlapping outbreak response measures identified in the US national FMD outbreak response plan^24^. Additionally, the model is flexible and allows for adjustment of select parameters, as was performed in the current study to explore the effect of preclinical transmission on simulated outbreaks. Similar to other disease spread models, this model is built upon assumptions concerning specific characteristics of the US production system for which it was designed. Extrapolation of the modeled output should therefore be done with caution. However, under the given circumstances, the outcome of the outbreak simulations included herein serve to emphasize the critical impact that the occurrence of disease transmission during the incubation phase may have on the magnitude of an FMD outbreak. Parameters for modeling of disease outbreaks are usually derived from meta-analyses of published experimental investigations that were not originally designed to assess disease transmission^25,26^. Bayesian methods offer an alternative approach to inferring epidemiologic parameters from transmission experiments, which can improve our understanding of the latent and infectious periods^6,12^. Regardless of the method used, in the absence of data explicitly describing actual disease transmission, different proxy measures must be used to define the transition between distinct stages of disease.

The use of proxies often results in the assumption that the onset of infectiousness is defined either by the first evidence of infection in a given individual (pathogen detection), or by the appearance of visible clinical signs of disease. In the current investigation, the proxy measure of high-threshold shedding was interpreted to be the most relevant predictor of transmissibility of FMDV. Onset of viremia was also correlated with infectiousness, which is likely due to the role of viremia in causing systemic  dissemination and shedding of virus. As demonstrated in this investigation, inappropriate use of proxy measures to define the onset of infectiousness in replacement of actual transmission data can lead to drastic misinterpretations of the transmission potential of infected animals. These misinterpretations may in turn affect FMD outbreak simulations, resulting in underestimation of outbreak size and severity, leading to unrealistic estimates of resource needs and misguided guidance on the appropriate application of control interventions. Specifically, underestimating the potential for disease transmission during the incubation phase may lead to wider dissemination of the outbreak than anticipated, and subsequent failure of reactive control interventions such as vaccination. Contrastingly, overestimating disease transmission can promote excessively aggressive countermeasures and lead to destruction of large numbers of healthy animals and associated economic losses. This may result in substantial animal welfare issues and added economic losses as products and animals from unaffected farms cannot enter the production chain. Appropriately balanced interventions are thus critical to effectively control disease spread while striving to maintain business continuity.

The current investigation demonstrated that the modeled onset of infectiousness in FMDV-infected pigs occurred approximately one day prior to appearance of clinical signs of disease, as was consistent with the primary descriptive data from these experiments^9^. This is consistent with earlier work which demonstrated FMD transmission during the incubation phase in pigs, lambs, and cattle^21^. However, one published work contradicts these findings by suggesting that FMD is unlikely to be transmitted before the onset of clinical disease^12^. Although there is limited published information on this subject, the consensus of available data suggests that subclinical transmission of FMD does occur, indicating that incubation is longer than latency and the resultant ω is greater than zero. This result was concluded based on the significant disparity between the durations of incubation and latency which indicated a distinct period of subclinical infectiousness that was demonstrated by experimentation and verified by modeling. Due to the limited group sizes and ubiquitous infection in groups to which transmission occurred, it was not possible to estimate R_0_ in the current investigation. However, previous investigations have estimated the basic reproduction ratio, R_0_, for FMDV within groups of non-vaccinated pigs in experimental settings to be as high as 30.74 (95% CI: 11.09–85.17)^21^ or 40 (95% CI: 21–74)^27^. The combination of a generally high R_0_ for within-group transmission of FMDV in pigs, and a positive value for ω as determined in the current study suggests that the potential for FMDV transmission during the incubation phase should be recognized when modeling FMD outbreak scenarios.

Our estimate for the proportion of preclinical transmission of FMDV that occurs in pigs was 12%, falling between values reported for SARS (θ < 11%) and smallpox (0 < θ < 20%)^11^. Fraser *et al*.^11^ identified a relationship between R_0_, θ, and the effectiveness of control interventions to bring an outbreak under control. As R_0_ and θ increase, control interventions must be highly effective, and multiple interventions are required in order bring the outbreak under control. Estimated values of R_0_ and θ for FMDV suggest that control of epidemics is dependent on using multiple, highly effective control interventions. Interestingly, these results may also suggest that isolation and contact tracing, if conducted nearly perfectly, could eventually be sufficient to prevent epidemic propagation. However, in the absence of significant technological advances, such interventions are unlikely to be implemented perfectly in livestock populations, and the timelines required for disease control could be much longer than when more interventions are enacted simultaneously.

The potentially profound consequences of FMDV incursions into regions previously free of FMD can, to a great extent, be attributed to the highly contagious nature of the virus. This was demonstrated in the current study by the maximum impact simulated example of infection of over 2,000,000 pigs when high ω was combined with a suboptimal outbreak response. Although FMDV can be transmitted via a multitude of both direct and indirect routes^28^, movement of infected animals has been identified as the most significant risk for dissemination of infection during the early phase of an outbreak^29^. This current investigation demonstrated the relevance of FMDV transmission during the incubation period in group-housed pigs. Furthermore, it was demonstrated that the duration of subclinical infectiousness had significant effects on spread and duration of simulated FMD outbreaks.

The data used for modeling of disease stage durations in the current study were derived from experiments in which pigs were infected with one specific FMDV strain, under experimental conditions. The virus strain and host criteria were chosen based upon extensive experience in our laboratory with these conditions and the assumption that the virus and conditions were representative of most virulent FMDVs. It is possible that disease dynamics in the field may differ due to strain-specific variations in virulence, as well as differences in animal age, health status, and housing conditions. Thus, it should be emphasized that the modeled output presented herein is based on experimental conditions, and represents our best estimate of what could be expected to occur under natural conditions in the field.

The findings presented herein demonstrate the importance of considering and elucidating the intricacies of key epidemiologic parameters, including preclinical infectiousness, the importance of understanding the relationships between proxy measures of disease status and infectiousness, and the subsequent value of incorporating these detailed parameters into disease spread models. Additionally, improved understanding of the influence of animal-level disease dynamics upon dissemination of FMD outbreaks may lead to improved approaches to surveillance and diagnostic testing to further refine control measures and maximize effectiveness while limiting undesired consequences for the agricultural industries.

## Methods

### Animal experiment

The data used in this current investigation were derived from an experimental trial designed to evaluate the onset of infectiousness in relation to the appearance of clinical disease in FMDV-infected pigs (Fig. 2). A detailed description of the experimental study and clinical findings has been published previously^9^. Animal experiments were carried out within BSL3-Ag facilities at Plum Island Animal Disease Center, New York. All procedures were carried out in accordance with guidelines specified within the associated experimental protocol (protocol 231-11-R), and were approved by the Plum Island Animal Disease Center Institutional Animal Care and Use Committee.

In brief, the study included 8 groups of 5 pigs of approximately 8–10 weeks of age (~25 kg), of which one group was infected with FMDV A_24_ Cruzeiro through simulated-natural inoculation^30^. The remaining 7 groups of pigs (contact groups 1–7) were sequentially exposed to the infected donor pigs through 8 hours of successive co-habitation within a designated isolation room (Fig. 2). After exposure to the donor pigs, contact-exposed pigs were moved into separate isolation rooms and were monitored for development of FMD. Samples collected were oropharyngeal swabs to assess virus shedding and blood samples to measure viremia. Clinical examinations and sample collection were done at 8–24 hour intervals after exposure. The choice to use FMDV A24 Cruzeiro for these experiments was based upon numerous previous experiments in our laboratory which had demonstrated that this strain was consistently virulent and transmissible in pigs^9,30–32^.

### Data analysis

#### Definitions

The end of latency for the donor pigs was defined as the beginning of the 8 hour contact exposure period during which the first successful transmission event occurred. It was not possible to attribute transmission events to specific individuals as donors and contact pigs were allowed to move freely within the exposure room. Thus, the earliest observed transmission event was used to define the transition from latent to infectious periods for all 5 donor pigs. The end of the incubation period for the donor pigs was determined for each pig individually by the first detection of vesicular lesions, which for all donor pigs coincided with detection of fever (rectal temperature ≥40 °C). FMDV shedding was defined by continuous detection of FMDV RNA in OPF. Viremia was defined by detection of FMDV RNA in serum.

#### Modeling of animal-level infection dynamics in FMDV-infected pigs

A Bayesian model was fitted to the data for the purpose of estimating the length of three distinct periods the donor pigs were expected to traverse: the latent, incubation, and infectious periods. A modeling approach was adapted from that published by Charleston *et al*.^12^. This model describes the relationship among the observed transmission successes and the unobserved latent, incubation, and infectious periods as well as the hyperparameters describing the distribution of those periods in the following way:

#### Likelihood

$$\begin{array}{llll}{\rm{L}}({\rm{\theta }},{\rm{E}},{\rm{C}},{\rm{I}}|{\rm{\delta }}) & = & {\rm{\Pi }}{\rm{j}}\,\{{{\rm{\Pi }}\mathrm{i\; p}}_{{\rm{ij}}}{{\rm{\delta }}}_{{\rm{ij}}}({\rm{1}}-{{\rm{p}}}_{{\rm{ij}}})1-{{\rm{\delta }}}_{{\rm{ij}}}\}\,{\rm{f}}({{\rm{E}}}_{{\rm{j}}},{{\rm{C}}}_{{\rm{j}}})\,{g(I}_{{\rm{j}}}), & \\ {\rm{where}}\,{{\rm{p}}}_{{\rm{ij}}} & = & 1-\exp \,(\,-\,{{\rm{\beta }}T}_{{\rm{ij}}}) & \\ {\rm{and}}:\,{{\rm{T}}}_{{\rm{ij}}} & = & {\rm{\max }}\,\{0,\,{\rm{\min }}\,{\{E}_{{\rm{j}}}+{{\rm{I}}}_{{\rm{j}}}-{{\rm{\tau }}}_{{\rm{ij}}}(0),{{\rm{\tau }}}_{{\rm{ij}}}(1)-{{\rm{\tau }}}_{{\rm{ij}}}(0)\}\} & {\rm{when}}\,{{\rm{E}}}_{{\rm{j}}}\le {{\rm{\tau }}}_{{\rm{ij}}}(0)\\ {{\rm{T}}}_{{\rm{ij}}} & = & {\rm{\min }}\,({{\rm{\tau }}}_{{\rm{ij}}}({\rm{1}})-{{\rm{E}}}_{{\rm{j}}},{{\rm{I}}}_{{\rm{j}}}) & {\rm{when}}\,{{\rm{\tau }}}_{{\rm{ij}}}(0) < {{\rm{E}}}_{{\rm{j}}}\le {{\rm{\tau }}}_{{\rm{ij}}}(1)\\ {{\rm{T}}}_{{\rm{ij}}} & = & 0 & {\rm{when}}\,{{\rm{E}}}_{{\rm{j}}} > {{\rm{\tau }}}_{{\rm{ij}}}(1)\end{array}$$

where:

i: indexes challenge event.

j: indexes donor pig.

δ_ij_: indicator for successful transmission in challenge i of donor j.

τ_ij_^(0)^/τ_ij_^(1)^: start/end time of challenge i of donor j.

p_ij_: probability of successful transmission in challenge i of donor j.

β: transmission rate.

T_ij_: time during challenge i for which donor j is infectious.

E_j_: latent period for donor j.

C_j_: incubation period for donor j.

I_j_: infectious period for donor j.

μ_E_, μ_C_, μ_I_, σ_E_, σ_C_, σ_I_, ρ_EC_: hyperparameters for latent, incubation, and infectious period prior distributions (in order; the means for the three periods, the standard deviations for the three periods, and the correlation between the latent and incubation periods).

α_E_, η_E_, α_C_, η_C_, α_I_, η_I_: hyperparameters for the means and standard deviations for the latent, incubation, and infectious period prior distributions (in order, the mean and standard deviation for the mean of the prior latent distribution, the mean and standard deviation for the mean of the prior incubation distribution, the mean and standard deviation for the mean of the prior infectious distribution).

The likelihood above describes the relationship between the observed transmission event data with the unobserved latent, incubation, and infectious periods, while the prior information (Table 3) was based on accumulated data from previous investigations carried out under similar conditions^30–33^, or were left diffuse in the absence of such information. These sources of information were combined and the posterior distribution over the parameters (latent, incubation, and infectious periods along with the means of the three periods) given the observed data (the outcomes of the transmission events) was estimated. This distribution is proportional to the product of the likelihood and the joint prior distribution for all of the parameters.

As the posterior conditional distributions for at least the latent period, infectious period, and the transmission rate were intractable, a numerical rather than an analytic approach was pursued. The model was coded using Just Another Gibbs Sampler (JAGS) software designed to perform Markov Chain Monte Carlo (MCMC) simulations^34^. This version of the model required two sacrifices: the latent and incubation periods could not be jointly lognormal, and the prior hyperparameter variance terms (σ_E_, σ_C,_ and σ_I_) were held fixed due to MCMC chain convergence issues as a result of unidentifiability issues. The result of the former being that the latent and incubation periods are assumed independent *a priori*.

The proportion of infection that occurs prior to onset of clinical disease, denoted as θ, is a function of the quantities estimated by the model described above. Using the JAGS version of the model adapted from Charleston *et al*.^12^ and assuming that latent and incubation periods are independent *a priori*, θ is described in as follows:

$${\rm{\theta }}\,=\frac{{\int }_{0}^{\infty }\{{\int }_{\tau }^{\infty }{f}_{C}(C)dC\}\{{\int }_{0}^{\tau }{f}_{E}(E)\{{\int }_{\tau -E}^{\infty }g(I)dI\}\}dEd\tau }{{\int }_{0}^{\infty }{\int }_{0}^{\tau }{f}_{E}(E)\{{\int }_{\tau -E}^{\infty }g(I)dI\}dEd\tau },$$

In addition to θ, we propose herein a distinct parameter (ω; omega) that allows more precise and direct inference about pre-clinical infectiousness to be made regardless of the total duration of the infectious period by representing the disparity between incubation and latency (Fig. 1).

$${\rm{\omega }}={\rm{C}}-{\rm{E}}$$

#### Estimating fluctuations of θ due to variations in duration of infectious period

As total duration of infectiousness was not experimentally evaluated in the current study, information about the length of the infectious period is drawn almost solely from the prior information. In order to evaluate the sensitivity of θ to variations in the total infectious period, θ was estimated as described above, but using incrementally increased integer values of infectious duration ranging from 1 to 14 days.

#### Modeling infection dynamics using different proxy measures to determine the onset of infectiousness

Four different proxy measures (Table 2) were evaluated for their ability to predict disease transmission. The outcome of modeling transmission using the defined proxy measures was compared to the standard model, which was based on confirmed transmission of FMDV to contact-exposed pigs (Confirmed Transmission Event (CTE) - Standard). The four evaluated proxies were defined as follows: (a) detection of FMDV RNA in serum, (b) detection of any FMDV RNA in OPF, (c) detection of FMDV RNA in OPF above a threshold of 6.5 log_10_ GCN/ml, which had previously been associated with successful transmission of FMDV^9^ (d) clinical signs of FMD. The data were input into a Bayesian model via five distinct indicator matrices, one for each measure of transmission, in which the number of rows equaled the number of contact animals-hours-post-inoculation combinations and the number of columns represented the five donor pigs. Each entry in the matrices was either a 1, if the donor animal met the criteria for successful transmission for the given transmission metric (Table 2) during exposure to the contact animal represented by that row, or a 0 if the donor animal did not meet the criteria for successful transmission given transmission metric at that time. For the CTE-standard transmission metric, which was defined by confirmed transmission to contact-exposed pigs, the vector for all donors was identical as the effect of individual donors on contact animal could not be determined as both sets of animals were exposed to one another in groups.

#### Simulation modeling of an FMD outbreak using estimated transmission parameters

The objective of the FMD outbreak simulations described herein was to evaluate the effect of altering the duration of the subclinical infectious period (omega; ω) on spread and duration of an outbreak of FMD in a US pig production sector. Estimates of disease stage durations were derived from modeling of animal-level infection dynamics (Table 1). The originally modeled output consisted of estimates for the durations of latent (E), incubation (C) and infectious (I) periods. The durations of the subclinical infectious period (ω = C − E) and clinical infectious period (I_C_ = I − C) were derived from these outputs. In addition to the baseline scenario (ω = 1 day), five omega values (0 day, 2 days, 3 days, 4 days, and 5 days) were subjectively selected to evaluate the effect on altering the duration of the subclinical infectious period on the spread and duration of simulated outbreaks. While choosing different omega values, the durations of latent and clinical infectious periods were kept constant (similar to baseline scenario) except for the scenario in which ω = 0 day of omega where the latent duration was set to 2 days (Supplementary Table S4). The Within-herd (WH) software version 0.9.7 available through the North American Animal Disease Spread Model^35^ was used to estimate herd-level parameters based on the modeled animal-level FMD disease stage durations. A spatial microsimulation model called the Farm Location and Agricultural Production Simulator (FLAPS) was used to generate a synthetic population file of 45,509 farms with 54,628,373 total pigs^36^ representative of pig production systems in the eastern United States (Supplementary Fig. S2). The US pig farms (with essential attributes for ISP such as identification number, herd size, type of farms, and Cartesian coordinates) located in the Great Lake, North East and South East regions were included in the model scenarios. About 72% (N = 45, 509) of the total pig farms in the United States are located in these regions. Amongst these farms, 31% were commercial farms with a median herd size of 2725 pigs (range: 100 to 60, 595) and 69% were small-scale enterprises with a median herd size of 6 pigs (range: 1 to 99). Farm-type specific movement parameters and contact rates were assigned to reflect differences in movements between commercial and small-scale enterprises. FMD outbreak simulations were performed using InterSpread Plus (ISP) version 6.0 model software^22^. The ISP is a state-transition, stochastic and spatial modeling tool for the simulation of FMD and other similar diseases^37^. Twelve FMD outbreak scenarios were developed in ISP representing optimal and suboptimal outbreak response conditions for each of 6 distinct values for the duration of subclinical infectiousness: 0 day, 1 day, 2 days, 3 days, 4 days, and 5 days of omega, respectively (Supplementary Table S4).

The unit of interest in the model was the individual farms. FMD epidemics were initiated from single farms. After exposure to FMDV, the susceptible farms were modeled to transit into latent, subclinically infectious, clinically infectious, and depopulated states. The spread of FMDV from an infected to susceptible farms was modeled to occur through direct contact, indirect contact, and local spread (Supplementary Note). The daily probability of transmission of FMD virus from infected farms to susceptible farm was calculated as the hypergeometric probability of shipping at least one infected animal off of an infected farm given the average herd size, shipment size, and the number of infected animals in a herd on a given day (Supplementary Fig. S3, Supplementary Note). Once an infected farm was detected, several control strategies were imposed simultaneously as is typically performed in response to outbreaks in FMD-free areas. Control strategies included zoning of control areas, tracing of animal movements, animal movement restrictions, depopulation of the infected farms, and surveillances as delineated in the national response plan for FMD^24^ (Supplementary Note). For each of the omega scenarios, two overall control strategies (optimal and suboptimal) were separately simulated. In the optimal control strategy, the detection of infected farms through passive surveillance was modeled to occur just after onset of clinical signs, which was further delayed by 14 days in suboptimal control category. Additionally, the delay in depopulation of detected farms was 3 (small farms) to 5 days (big farms) in the optimal control category, which was delayed by 7 more days to represent the suboptimal control. The major outputs parameters were outbreak size (number of infected farms and pigs), epidemic duration (days from onset of infection to end of epidemic), time between onset of infection to detection, and daily new infected farms due to each of the incorporated omega values. The median and interquartile range of outbreak size and epidemic duration were reported. The data analyses were performed using SAS (SAS Institute Inc., NC, USA, 2017) and Microsoft Excel (Microsoft Excel, Redmond, Washington, 2017). The Kruskal-Wallis test with Bonferroni corrections was performed for multiple group comparisons for the outcomes from various omega values. A p-value of ≤0.05% was considered for statistical significance.

## Supplementary information

Quantitative impacts of incubation phase transmission of FMDV_Supplement

## Data Availability

All data generated or analyzed during this study are included in this published article and its Supplementary Information Files.
