# Supplementary material for: Quantitative impacts of incubation phase transmission of foot-and-mouth disease virus
Source: Sci Rep. 2019 Feb 25;9:2707. doi: 10.1038/s41598-019-39029-0 (PMC6389902; doi:10.1038/s41598-019-39029-0)
Supplement: Supplementary file 1 — Quantitative impacts of incubation phase transmission of FMDV_Supplement [file 41598_2019_39029_MOESM1_ESM.pdf]

Supplementary material

Quantitative impacts of incubation phase transmission of foot-and-mouth disease virus

Jonathan Arzt, Matthew A. Branan, Amy H. Delgado, Shankar Yadav, Karla I. Moreno-Torres, Michael J. Tildesley, Carolina Stenfeldt

*Corresponding authors: Jonathan.Arzt@ARS.USDA.GOV, Carolina.Stenfeldt@ARS.USDA.GOV

Supplementary Figure S1.

Progression of infection in the group of donor pigs (n=5) defined by detection of FMDV RNA in oropharyngeal fluid (**A**) and serum (**B**) as well as appearance of vesicular lesions characteristic for FMD (**C**). Donor pigs were infected with FMDV serotype A Cruzeiro by intra-oropharyngeal inoculation. Clinical examinations and collection of OPF was done at 8-hour intervals from 0 to 64 hours post inoculation (hpi). Serum was collected at 0, 16, 24, 48 and 64 hpi. Whiskers indicate range, horizontal lines are medians and the boxes mark the 25th and 75th percentiles.

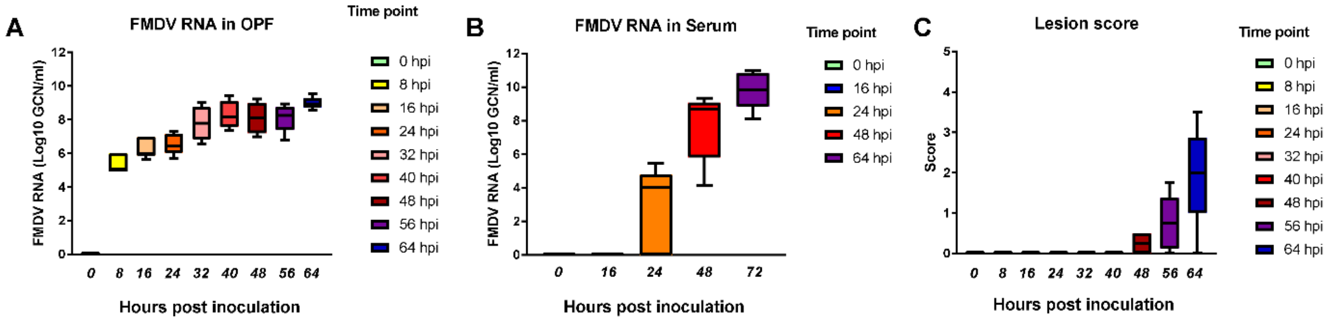

Supplementary table S1. Estimates of the proportion of subclinical transmission (θ) based on the estimated duration of the subclinical infectious period (ω) and incrementally increased duration of total infectiousness.

Mean Infectious Period duration (days)	Median θ	95% CI
**1**	0.540	0.053 -1.30
**2**	0.440	-0.0027-1.20
**3**	0.290	0.033-0.65
**4**	0.220	-0.025-0.61
**5**	0.150	0.0056-0.37
**6**	0.160	0.014-0.39
**7**	0.130	0.0045-0.30
**8**	0.110	0.009-0.24
**9**	0.110	0.012-0.26
**10**	0.083	0.0082-0.20
**11**	0.083	0.0059-0.19
**12**	0.069	-0.00033-0.16
**13**	0.067	0.0043-0.16
**14**	0.055	0.002-0.14

Supplementary Table S2. Posterior medians for model estimates when using different proxy measures to define the onset of infectiousness in donor pigs.

Transmission metric	Symbol	Confirmed Transmission Event (CTE) - Standard		FMDV RNA in Serum		FMDV RNA in OPF (baseline)		FMDV RNA in OPF (>6.50 log10 GCN/ml)		Clinical Signs in Donors	
		median	95% CI	median	95% CI	median	95% CI	median	95% CI	median	95% CI
**Latent period**	E	27	(24, 30)	30	(29, 31)	13	(11, 15)	20	(19, 21)	50	(48, 53)
**Incubation period**	C	48	(29, 71)	48	(29, 74)	48	(28, 72)	48	(30, 73)	49	(28, 72)
**Infectious period**	I	180	(110, 270)	180	(110, 270)	180	(110, 280)	180	(120, 260)	180	(110, 270)
**Subclinical infectious period**	ω	21	(1.1, 45)	19	(-0.76, 44)	35	(15, 59)	29	(9.8, 53)	-1.20	(-22, 22)
**Proportion of total infectious period that is subclinical**	θ	0.12	(0.00083, 0.27)	0.10	(-0.011, 0.26)	0.19	(0.067, 0.36)	0.16	(0.041, 0.32)	-0.0062	(-0.13, 0.14)
**Mean length of latent period**	μ_E	27	(25, 30)	29	(27, 31)	13	(11, 15)	19	(18, 21)	49	(47, 52)
**Mean length of incubation period**	μ_C	48	(29, 74)	48	(30, 73)	48	(30, 73)	49	(30, 72)	49	(28, 71)
**Mean length of infectious period**	μ_I	180	(110, 280)	180	(100, 260)	180	(110, 270)	180	(120, 270)	180	(120, 270)
**Transmission rate**	β	27	(13, 57)	52	(19, 98)	36	(15, 68)	46	(14, 91)	32	(13, 66)

Supplementary note. Simulation modeling of an FMD outbreak using estimated transmission parameters.

FMD outbreak simulations were performed using InterSpread Plus (ISP) version 6.0 model software. Out of total swine farms included in the model, 31% were commercial and 69% were small-scale enterprises. Farm-type specific movement parameters and contact rates were assigned to reflect differences in movements between commercial and small-scale enterprises. Animal movements (direct contacts) for breeding pigs, weaned pigs, feeder pigs, and pigs from commercial farms, small-scale enterprises, and markets were simulated based on contact rates and destination farm type probabilities for each production type (Supplementary Table S5). For commercial farms, indirect contacts were classified as high risk or low risk contacts, based on the potential for viral contamination and animal contact. The high risk contacts included veterinarians, customers, dealers, employees with livestock at residence, extension agents, livestock haulers including those used for dead box pick-ups, and manure haulers. Low risk indirect contacts included commodity/feed trucks, shared equipment, drivers of livestock haulers, nutritionist, feed company consultants, other vehicles such as postal deliveries, and visitors. Low risk indirect contacts are assumed to follow the biosecurity protocols of the commercial farm, leading to a reduced risk of disease transmission to animals. In contrast, given the variability in biosecurity on small-scale enterprises and dealers, all indirect contacts are assumed to have the same level of transmission risk for these farm types. The risk of disease transmission from movements to slaughter (including culled sow movements) was not explicitly modeled in these scenarios. The frequency of direct and indirect contacts, distance traveled, and destination probabilities were derived from data collected as part of the National Animal Health Monitoring System (NAHMS) Swine 2012 national survey and the NAHMS Swine 2007 Small-Enterprises Study (Supplementary Tables S6 - S7). Local area spread, which represents unknown or difficult to trace mechanisms, such as vectors or insects, which may act over short distances, was allowed to occur from infected to susceptible farms over a distance of 4km.

The daily probability of transmission from direct contacts between infected and susceptible farms was calculated as the hypergeometric probability of shipping at least one infected animal, given the average herd size, shipment size, and the number of infected animals in a herd on a given day. The daily prevalence of infectious pigs in the herd was estimated for each of the omega values and farm types (commercial farms, small farms, and dealers) using the WH model. The daily prevalence of infectious pigs was multiplied by the median herd size to estimate the daily number of infected pigs in the herd. The daily probability transmission for direct contacts, indirect (high risk or low risk) contacts and local spread are summarized in the Supplementary Figure S3 and Supplementary Table S8. Detection of the first infected farms was assumed to occur through passive surveillance dependent on farm workers, veterinarians or owners observing animals with clinical signs. Following the first detection of the disease, the probability of detection by farmers and veterinarians, through direct or indirect tracing, and surveillance was increased from day 1 (5%) to day 11 (99%) (Supplementary Table S9) in order to reflect the

heightened awareness of the disease that would occur following an outbreak detection. Detection of an infected farm was followed by creation of two radial zones (10km-inner and 20km-outer) around each of the detected farms. Active surveillance activities were initiated within 20 km surveillance zones around infected farms, which allow for random selection and testing of farms within the control zone. Tracing was initiated to identify farms which were in direct or indirect contact with the infected farms. The time required to complete a trace (in days) for all animal movements and low risk indirect contacts was set as BetaPert (0,1,2). In addition, the model assumed that 20% of movements and low risk indirect contacts were forgotten by farmers. For the high risk indirect contacts, the tracing delay (in days) was BetaPert (0,2,3) with a higher probability of movement (75%) forgotten by farmers. Movement restrictions were applied to all farms within a 10km radial zone of infected or detected farms, and the probability of movement restricted was 0.95 and 0.25 for direct and indirect movements, respectively. All pigs on infected farms were depopulated at the rate of 15 farms per day.

Supplementary Table S3*. Outcome of FMD outbreak simulations using different durations of subclinical infectiousness (ω) and optimal versus suboptimal outbreak response strategies.

Omega (days)	Epidemic duration (days)	Time to detection (days)	Number of infected farms	Number of pigs on affected farms
**Optimal response**				
**0**	39 (18, 36)	12 (10, 14)	32 (8, 78)	173536 (57518, 418455)
**1**	48 (22, 73)	12 (10, 14)	57 (13, 124)	277930 (76265, 642444)
**2**	59 (28, 94)	14 (13, 16)	71 (15, 153)	364836 (90319, 762832)
**3**	70 (39, 103)	16 (15, 18)	83 (23, 164)	428499 (123167, 821069)
**4**	86 (51, 123)	18 (17, 20)	106 (35, 201)	533881 (186842, 972854)
**5**	93 (59, 138)	20 (19, 22)	120 (43, 230)	596962 (224484, 1060379)
**Suboptimal response**				
**0**	88 (73, 112)	27 (25, 28)	415 (297, 537)	1864569 (1384932, 2374743)
**1**	101 (82, 124)	27 (25, 28)	581 (428, 752)	2529481 (1903770, 3216530)
**2**	119 (96, 146)	29 (27, 31)	604 (450, 778)	2586261 (1976090, 3206650)
**3**	133 (108, 165)	31 (29, 33)	619 (463, 800)	2617873 (2031086, 3252107)
**4**	149 (119, 185)	33 (31, 35)	648 (476, 832)	2691168 (2040470, 3338918)
**5**	163 (131, 205)	34 (33, 37)	635 (453, 843)	2622455 (1928637, 3291677)

*All values are presented as median (25th, 75th)

Supplementary Figure S2. Geographic region used for simulation modeling.

A synthetic population file of 45,509 farms with a total of 54,628,373 pigs, representative of pig production systems in the eastern United States was created using a spatial microsimulation model called the Farm Location and Agricultural Production Simulator (FLAPS).

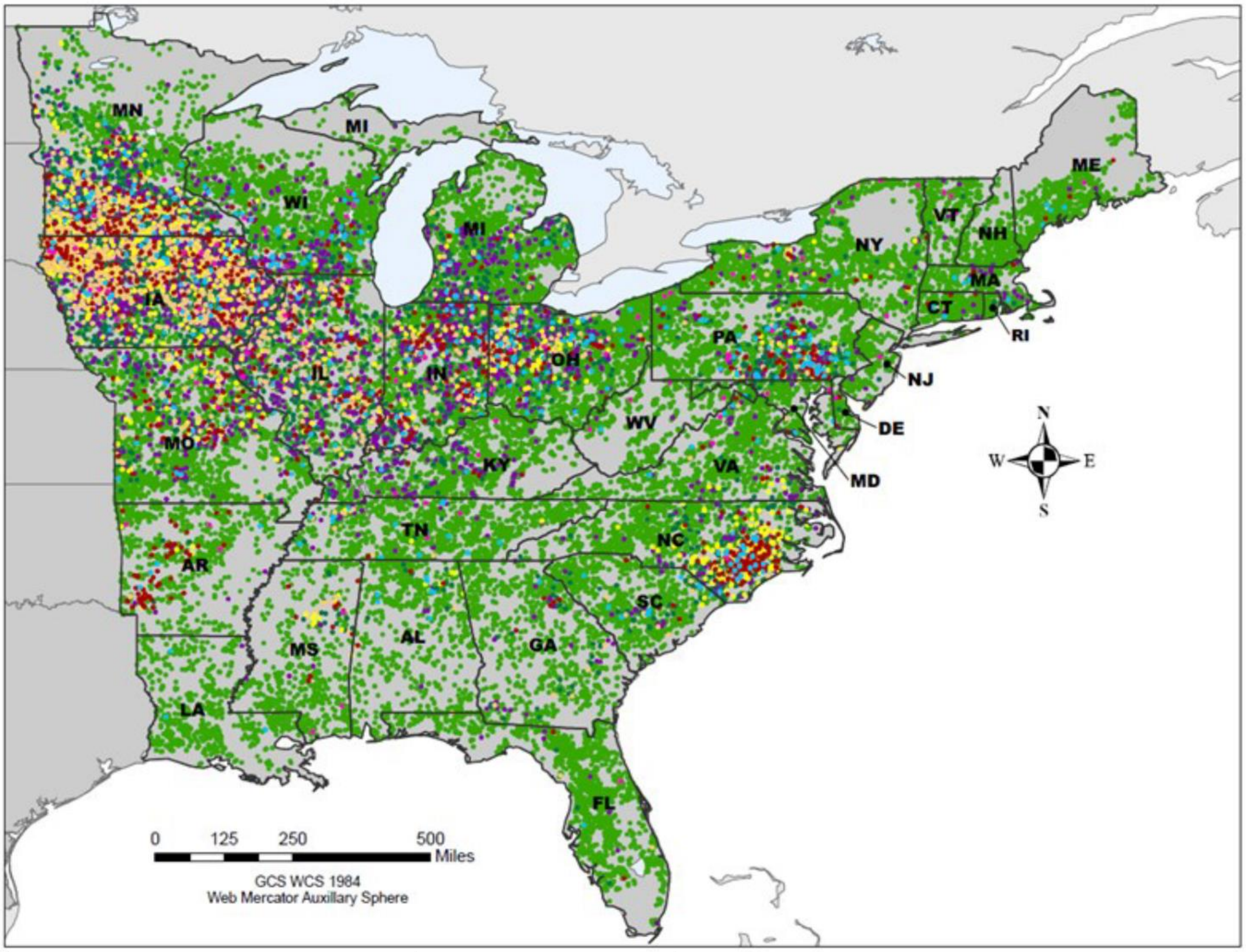

Operations type	Percentage of total swine farms	Herd size		
		Min	Median	Max
Dealer	0.2%	6	53	81
Small-scale swine enterprises	69%	1	6	99
Swine nursery	3%	108	3937	57941
Swine grow-finisher	16%	100	3304	60595
Swine farrow-to-wean	2%	101	2748	45208
Swine farrow-to-feeder	0.8 %	100	342	14692
Swine farrow-to-finish	7%	100	642	45477
Swine Other	2%	100	3452	44047

Supplementary Figure S3. Probability of transmission of FMDV from infected to susceptible swine herds due to (A) direct contact, (B) indirect (high risk and low risk) contact and movements from and to market. The probability of transmission due to direct contact was estimated for all six omega values for commercial swine operations, dealers and swine small-scale enterprises. The probability of transmission of FMDV estimated for the dealers and swine small-scale enterprises was 100% from day 1.

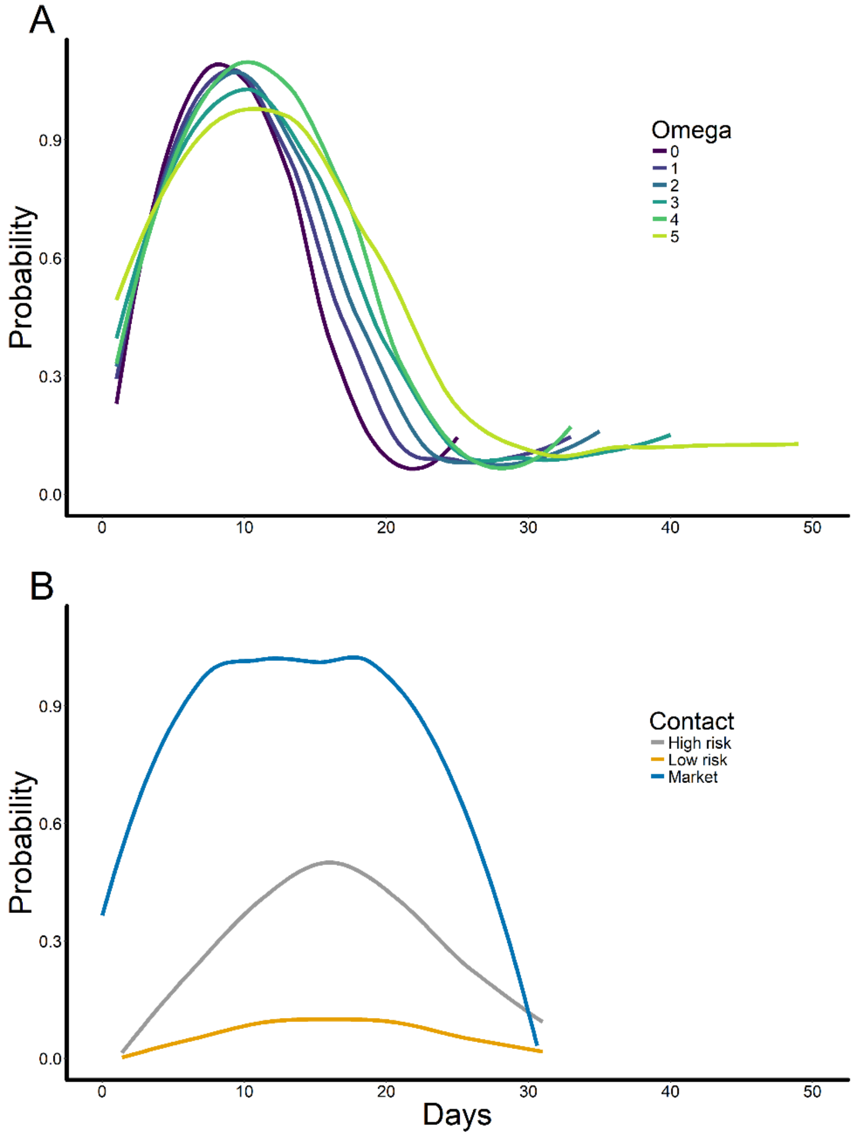

Supplementary Table S4. Animal-level and herd-level parameters incorporated in the Within-herd (WH) and InterSpread Plus (ISP) models.

**Parameters**		**Scenarios**						**References**
		**1**	**2**	**3**	**4**	**5**	**6**	
Animal-level (WH-model) parameters	*Latent period (day)	1	2	1	1	1	1	Baseline (scenario 1) values were estimated
	*Subclinical infectious: ω (day)	1	0	2	3	4	5	
	*Clinical infectious (day)	7	7	7	7	7	7	
	Immune Period (day)	Gaussian(360,90)						(USDA, 2013)
	Population size	*Commercial farm:* Negbinom(1, 0.00025863),<br>*Dealer:* Negbinom(23,0.30361),<br>*Small farms:* Negbinom(1,0.080395)						Derived from data
	Adequate exposure per day	Weibull(5,200)						(USDA, 2013)
	Initially latently infected pig	1						
**Herd-level (ISP model) parameters	Incubation period (day)	2	2	3	4	5	6	Estimated
	Total infectious duration (day)	12	12	13	14	15	16	Estimated

*The latent, subclinical infectious (ω) and clinical infections durations were from the animal-level estimates, and these estimates were incorporated as the constant value in the WH model.

** The herd-level estimates (incubation duration and total infectious durations) were estimated from the animal-level estimates using the WH-model software. The herd-level estimates were input parameter values for the ISP model.

Supplementary Table S5. Contact rates and destination probabilities of direct swine movements* within the US.

Movement type	Source farm types	Contact rate (movement /farm/day)	Destination farm type	Destination Probability
Breeding pigs (sow, gilt, boar)	Swine other (large) Swine other (small)	0.0413	Farrow to feeder (large)	0.0126
			Farrow to feeder (small)	0.067
			Farrow to finish (large)	0.2708
			Farrow to finish (small)	0.4239
			Farrow to wean (large)	0.1446
			Farrow to wean (small)	0.0611
			Small-scale enterprises	0.02
	Grower finisher (large) Grower finisher (small)	0.0015	Farrow to feeder (large)	0.0126
			Farrow to feeder (small)	0.067
			Farrow to finish (large)	0.2708
			Farrow to finish (small)	0.4239
			Farrow to wean (large)	0.1446
			Farrow to wean (small)	0.0611
			Small-scale enterprises	0.02
Weaned pigs	Farrow to wean (large) Farrow to wean (small)	0.4068	Nursery (large)	0.8254
			Nursery (small)	0.1546
			Small-scale enterprises	0.02
Feeder pigs	Farrow to finish (large) Farrow to finish (small)	0.0209	Grower finisher (large)	0.7397
			Grower finisher (small)	0.2603
	Farrow to feeder (large) Farrow to feeder (small)	0.1049	Grower finisher (large)	0.7297
			Grower finisher (small)	0.2503
			Small-scale enterprises	0.02
	Nursery (large) Nursery (small)	0.0868	Grower finisher (large)	0.7297
			Grower finisher (small)	0.2503
			Small-scale enterprises	0.02
Small-scale enterprises	Small-scale enterprises	0.0023	Small-scale enterprises	1
Market	Market	0.001	Farrow to feeder (large)	0.0267
			Farrow to feeder (small)	0.0535
			Farrow to wean (large and small)	0.0267
		0.004	Farrow to finish (large)	0.1337
			Farrow to finish (small)	0.2941
	Market	0.0077	Dealer	0.4386

*Movements to slaughter were not modeled

Supplementary Table S6. Indirect contact rates amongst U.S swine farms. The frequency of indirect contacts were derived from data collected as part of the National Animal Health Monitoring System (NAHMS) Swine 2012 national survey and the NAHMS Swine 2007 Small-Scale Enterprises Study

Operations	High risk movements[1]	Low risk movements[2]
Large swine operations	Poisson(2.214)	Poisson(1.239)
Small swine operations	Poisson(0.3387)	Poisson(0.2119)
Small-scale enterprises[3]	Poisson(0.094)	Poisson(0.094)
Dealers	Poisson(0.0162)	Poison(0.0162)

[1]Included veterinarians, customers, dealers, employees with livestock at residence, extension agents, livestock haulers including those used for dead box pick-ups, and manure haulers

[2] Included commodity/feed trucks, shared equipment, drivers of livestock haulers, nutritionist, feed company consultants, other vehicles such as postal deliveries, and visitors. Low risk indirect contacts are assumed to follow the biosecurity protocols of the commercial farm, leading to a reduced risk of disease transmission to animals.

[3] Given the variability in biosecurity on small-scale enterprises and dealers, all indirect contacts are assumed to have the same level of transmission risk for these farm types.

Supplementary Table S7. Probability of movement of shipments by distance band for commercial and small-scale swine operations, dealers and markets in the Eastern United States.

Distance (meters)	Probability of shipment								
	Grow to finish		Farrow to wean		Farrow to feed, Finisher, Nursery		Small-scale enterprises	Dealer/Market	
	Great Lake	Other region	Great Lake	Other region	Great Lake	Other region	All region	Distance (meters)	All region
0	0	0	0	0	0	0	0	0	0
29000	0.01	0.01	0.207	0.069	0.422	0.185	0.3	100000	0.83577
73000	0.57	0.485	0.317	0.004	0.166	0.154	0.5	200000	0.1129
161000	0.2	0.216	0.301	0.397	0.374	0.491	0.15	300000	0.02311
322000	0.05	0.01	0.122	0.085	0.008	0.003	0.04	400000	0.01195
2900000	0.17	0.279	0.053	0.445	0.03	0.167	0.01	500000	0.01259
								600000	0.00299
								700000	0.00027
								800000	0.00037
								900000	0.00005

Supplementary Table S8. Probability of transmission of FMDV due to local spread from infected but not detected farms, detected but not depopulated farms, and depopulated but disposal incomplete farms. The estimations were derived from Sanson et al., 2006[1].

Farm states	Distance (km)	Probability of transmission (POT)				
Undetected	1	0	0.07	0.12	0.12	0.12
	2	0	0.02	0.003	0.004	0.004
	3	0	0	0.001	0.001	0.001
	4	0	0	0	0	0
Detected but not depopulated	1	0	0.0035	0.006	0.006	0.006
	2	0	0.001	0.0013	0.002	0.002
	3	0	0	0.005	0.0005	0.0005
	4	0	0	0	0	0
Depopulated but disposal not completed	1	0	0.00175	0.003	0.003	0.003
	2	0	0.005	0.0075	0.001	0.001
	3	0	0	0.00025	0.00025	0.00025
	4	0	0	0	0	0

[1] R. Sanson, M. Stevenson, N. Moles-Benfell **Quantifying local spread probabilities for foot-and-mouth disease** Proceedings of the 11th International Symposium on Veterinary Epidemiology and Economics, Cairns Convention Centre, Cairns, Australia (2006)

Supplementary Table S9. Probability of detection of FMDV infected pigs at the herd-level in various swine operations of the United States incorporating surveillance activities. It specifies the probability of a farm being detected per unit time.

**Swine operations**	**Surveillance phases / zones**	**Days**										
		**1**	**2**	**3**	**4**	**5**	**6**	**7**	**8**	**9**	**10**	**11**
Small-scale enterprises	Silent	0	0.05	0.1	0.4	0.6	0.8	0.99				
Small farms		0	0.05	0.1	0.2	0.49	0.74	0.9	0.99			
Large farms		0	0.05	0.05	0.05	0.1	0.2	0.3	0.5	0.7	0.9	0.99
Small-scale enterprises	Response	0	0.05	0.03	0.6	0.9	1					
Small farms		0	0.05	0.05	0.3	0.6	0.9	1				
Large farms		0	0.05	0.05	0.05	0.3	0.4	0.6	0.8	0.9	1	
All	Tracing and 10km & 20km	0	0	0.25	0. 5	0.5	0.75	0.95	0.95	0.95	0.99	
